# Contextual influences on risk-taking in children and adults

**DOI:** 10.3389/fnbeh.2025.1644777

**Published:** 2025-10-22

**Authors:** Penelope Lacombe, Klaus Zuberbühler, Christoph D. Dahl

**Affiliations:** ^1^Institute of Biology, University of Neuchâtel, Neuchâtel, Switzerland; ^2^School of Psychology and Neuroscience, University of St Andrews, St Andrews, United Kingdom; ^3^Graduate Institute of Mind, Brain and Consciousness, Taipei Medical University, Taipei City, Taiwan

**Keywords:** behavioral economics, risk-taking behavior, cognitive biases, decision-making, risk preference, framing effect

## Abstract

Human risk-taking is well known to be influenced by context-dependent factors. In a previous study, we demonstrated that non-human primates similarly exhibit contextual risk-preference: two species of great apes showed risk-prone or risk-neutral choices depending on the manner in which risk was presented. Here, we applied the same experimental paradigm to human participants across different age groups using a computerized online interface. Consistent with the findings in great apes, we observed shifts in risk preference contingent on the experimental context, with these effects particularly pronounced in children. In a subsequent experiment, we explored potential cognitive mechanisms underlying this preference shift, identifying a general propensity for exploration and framing effects as promising explanatory factors common to both humans and animals.

## 1 Introduction

The economic literature proposes several models to predict human decision-making. One foundational model is Expected Utility Theory, a normative model that states that individuals make rational decisions and try to maximize utility (Harrison et al., 1945). A more nuanced alternative is Cumulative Prospect Theory (Kahneman and Tversky, 1979) which posits that people evaluate outcomes relative to a reference point (also called the *status quo*), while overweighting small probabilities (e.g., winning the lottery) and underweighting large probabilities (e.g., neglecting insurance for major risks). This “framing effect” described by the Cumulative Prospect Theory leads to apparently irrational behavior, with risk-averse choices if decision is framed positively (i.e., in the context of gains) and risk-prone choices is decision is framed negatively (i.e., in context of loss).

Although Prospect Theory is a descriptive model that does not explain, for instance, how and why reference points are used to make decision, this model is empirically well supported in adult humans (Kühberger, 1998). Research on children showed that their attitude toward risk was somewhat similar to that of adults: consistent with Prospect Theory, children as young as 5 years-old use expected value to guide their decision (Schlottmann and Anderson, 1994), and tend to be more risk-prone in the loss domain than in the gain domain (Schlottmann and Tring, 2005).

Risk-sensitivity was also studied by behavioral ecologists to understand foraging strategies in animals, which lead to the so-called energy budget rule. This energy budget rule states that animals are risk-sensitive and make their foraging decisions based on the probability to avoid starvation, by comparing the energy budget provided by a constant or variable option to their survival threshold (Caraco, 1980; Stephens, 1981). This risk-sensitive theory is a normative model than could explain the origin of reference points (minimal acceptable requirements, or need) in Prospect Theory. First developed to explain non-human behavior, experimental findings showed that there is also evidence for an energy budget rule in humans (Mishra and Fiddick, 2012; Wyszynski and Diederich, 2022).

Thus, Prospect theory and risk-sensitive theory are well supported in animals and humans, showing a similarity between humans and animal decision-making in risky contexts. For instance, capuchin monkeys and orang-utans, like humans, distort probabilities by overweighting small probabilities and underweighting large ones, consistent with predictions from Cumulative Prospect Theory (Pelé et al., 2014). Similarly, bonobos and chimpanzees exhibit risk-prone behaviors when options are framed as losses and risk-averse behaviors when the same options are framed as gains, again aligning with Cumulative Prospect Theory (Krupenye et al., 2015). Framing effect in non-human primates was also documented in capuchin monkeys (De Petrillo et al., 2015), macaques (Yamada et al., 2013), orang-utans (Pelé et al., 2014), and gorillas (Leinwand et al., 2020).

However, the literature on risk-taking does not present a uniform picture; in the animal literature, findings are often inconsistent across species, such as macaques [ Yamada et al. (2013) find macaques risk-averse while Pelé et al. (2014) find them risk-prone] and great apes [risk-aversion described in Rosati and Hare (2013) and risk-proneness described in Haun et al. (2011)]. Instead, risk preferences seem flexible and highly context-dependent, even within the same species, seemingly influenced by minor factors like variations in inter-trial intervals, reward type, or reward magnitude (Heilbronner and Hayden, 2013; De Petrillo and Rosati, 2021). The existence of a framing effect in animals is also discussed (Kanngiesser and Woike, 2016) as studies show an inconsistency of framing across species [risk-seeking behavior in gain framed-experiment is described in Krupenye et al. (2015), while risk-aversion in gain-framed experiments in Lakshminarayanan et al. (2011) for instance].

Similarly, in the human literature, risk-preference is variable across studies, as several factors can affect behavior [age (Levin and Hart, 2003), time constraint (Diederich et al., 2020), use of within-participants or between-participants design (Appelt et al., 2011), differences in subject attention levels (Orquin et al., 2018), level of perceived need (Wyszynski and Diederich, 2022), type of experimental task (Diederich et al., 2018),…]. Previous research (Crosetto and Filippin, 2016) investigated the heterogeneity of results in the human economic literature and the low correlation observed between risk levels measured with different tasks. They suggest that risk is difficult to measure without error, and that the different tasks classically used to measure it may trigger different mechanisms [for instance, an increased regret (Loomes and Sugden, 1982) or a certainty effect (Andreoni and Sprenger, 2011) may be caused by the use of a safe option].

Thus, a key challenge to establishing robust evolutionary and ontogenetic accounts of economic decision-making is the considerable variation in experimental designs across studies, especially across species. Animal studies typically involve numerous trials with short inter-trial intervals and small food or juice rewards, conditions that may diminish the perceived stakes of risk (Heilbronner and Hayden, 2013). Indeed, when satiety levels are controlled, monkeys’ responses become more comparable to those observed in humans (Yamada et al., 2013).

Another fundamental difference lies in the information available: humans are usually explicitly informed about the probabilities and outcomes (e.g., “Option A yields a 50% chance of winning 2 Euros”), whereas animals must infer reward contingencies through exploration and experience. This ‘description-experience gap’ (Hertwig and Erev, 2009) can significantly affect decision-making, as small probabilities tend to be overestimated when described but underestimated when learned through experience (Hertwig and Erev, 2009; Erev et al., 2010). These distortions may arise from factors such as sampling bias, estimation errors (Fox and Hadar, 2006), and recency effects (Hertwig et al., 2004). This description-experience bias also seem to affect behaviors even when rare events are not considered, as (Ludvig and Spetch, 2011) showed that experiments from experience induced a reversion of the framing effect: subjects expressed higher levels of risky choices in the gain domain than in the loss domain in experience-based experiments, which contradicts classical Prospect Theory.

More recent works tried to compensate the heterogeneity of protocols used to study risk behavior in humans and animals by adapting human-tasks to animals (Ludvig et al., 2014) or animal-tasks to humans (Hayden and Platt, 2009).

Similarly, ontogenetic research is often hindered by incompatible experimental designs, including differences in stimuli or reward types between adults and children designs. For example, food or toys are common rewards for children, whereas adults usually receive monetary incentives—yet money tends to increase risk aversion compared to tangible rewards like food or prizes (Rosati and Hare, 2016). Crucially, described probabilities may be difficult to use to young children via linguistic instructions alone. Instead, they are typically conveyed through tangible representations, such as spinner-wheel segment sizes (Harbaugh et al., 2002) or card frequencies (Kerr and Zelazo, 2004), making difficult to compare results with adult literature where description-based designs are often used.

These considerations from both ontogenetic and phylogenetic research underscore the urgent need for standardized experimental designs when investigating the origins of economic decision-making. However, only a few studies [e.g., (Broihanne and Dufour, 2018; Rivière et al., 2018; Roig et al., 2022; Haux et al., 2023),…] have facilitated direct cross-age or cross-species comparisons, presenting a major obstacle to theoretical integration and progress.

In a previous work, in order to address between-studies results in the non-human literature, we compared two experimental designs (a “single-cup” and a “multi-cup” design) used to assess risk-preference in great apes [gorillas and orang-utans (Lacombe et al., 2022)] and found that individuals chose between a safe and a risky option according to the expected value of both options, but that the experimental design interacted the rational comparison of expected values. The single-cup design employed, following (Heilbronner et al., 2008), required individuals to choose between a safe cup providing a known and constant reward, and a risky cup yielding a variable reward. As anticipated from previous research (Heilbronner et al., 2008; Rosati and Hare, 2013; Keupp et al., 2021), this design elicited risk-averse choice. In contrast, our multi-cup design, adapted from Haun et al. (2011), presented subjects with a choice between a safe cup with a known reward and a tray of risky cups, only one of which contained a known reward. As expected based on Haun et al. (2011), this design led to risk-proneness.

This shift in risk-preference between the two designs, even though they had the same economic parameters (same expected value, same probability to win and same outcome amounts) suggested the influence of one or several factors, such as the description-experience gap (the single-cup design being an experience-based design and the multi-cup design an experience-based one), differences in exploration-exploitation strategies (with a risky option in the multi-cup design that could trigger more exploration strategies), or differences in framing (a common mechanism underlying contextual preferences).

To enable direct comparisons with the primate findings, we used the same tasks (single-cup design and multiple-cup design) and adapted them to human subjects, to test human adults and children. Our aims were twofold: (1) to determine whether humans exhibit similar risk preference shift between the single-cup and the multi-cup design, and (2) to investigate which factors could influence their decision-making. To achieve this, we developed a computerized version of the task used in Lacombe et al. (2022), preserving the same quantitative contingencies, such as probabilities of risky options and reward values. If the economic decision-making observed in great apes reflects an evolutionarily ancient predisposition, we expected to observe comparable effects in modern humans (Rangel et al., 2008). In the second phase of the study, we sought to investigate the nature of the factors that might underlie the observed shifts in risk preference.

We focused on two promising candidates to explain the risk-preference shift: the description-experience gap (Hertwig and Erev, 2009) and the exploration-exploitation dilemma (Cohen et al., 2007). As outlined earlier, the description-experience gap refers to distortions in probability and task perception depending on whether probabilities are learned through experience (as in the single-cup design) or explicitly described (as in the multiple-cup design). To test this, we modified the single-cup design by providing participants with explicit information about the probability of winning for the risky option, thereby removing the need to learn probabilities through sampling. If the description-experience gap is responsible for the risk-preference shift observed between the single- and multiple-cup designs, this modification should reduce or even eliminate the shift.

Second, concerning the exploration-exploitation dilemma, we sought to determine whether differences in exploration strategy could explain the risk-preference shift. In the single-cup design, the risky option is presented as a single cup, while in the multiple-cup design, it appears as a tray of cups—an arrangement that may encourage greater exploration due to its increased salience. To test this hypothesis, we modified the multiple-cup design by replacing the tray with a single cup, while providing participants with explicit information about the potential reward and its associated probability. If exploration strategies underlie the risk-preference shift, this modification should reduce or eliminate the difference in behavior between the two designs.

## 2 Materials and methods

### 2.1 Subjects

#### 2.1.1 Adults

The experiment was conducted online via a dedicated website, which was promoted on social media (i.e., Facebook). Out of *N* = 130 participants who created an account on the website and initiated the experiment, 90 completed the first part (39 females, age from 20 to 87 years, with a mean of 34 years) and 40 did not complete the experiment (18 females, age from 17 to 69, with a mean of 31 years). Although the website was available in both French and English to maximize participant diversity, all 90 subjects were French. Most participants (*n* = 50) were employed in the science sector (e.g., researchers, engineers). The remaining participants were in the administration or health sectors (*n* = 17), the private sector (*n* = 7) or they were unemployed, students or retired (*n* = 16). Six months after the completion of the first phase, participants were invited to take part in the second phase of the experiment. A total of 37 subjects (15 females) completed the second phase, with ages ranging from 24 to 87 years (mean = 33 years).

#### 2.1.2 Children (5–11 years)

The experiment was conducted online, with recruitment by means of social media and ZolliGumper, a child group affiliated with Basel Zoo).^1^ The parents of the children received emails inviting them to participate. Participants were informed that upon completing the entire experiment, they would receive a 15 €/CHF gift card. The website was available in French, English and German; all children recruited via Facebook were French-speaking, while those via ZolliGumper were all German-speaking. A total of 62 children began the experiment, 39 completed the first part (21 females, with an average age of 8 years) and 23 did not complete the experiment (9 females, with an average age of 7 years). Among the completers, 24 have been recruited via Facebook and 15 via the ZolliGumper group. The participants ranged in age from 5 to 11 years, with a mean of 8 years-old (5 years: *n* = 5; 6 years: *n* = 7; 7 years: *n* = 2; 8 years: *n* = 6; 9 years: *n* = 12; 10 years: *n* = 6, 11 years: *n* = 1). Six months after the completion of the first phase, children were invited to participate in the second phase of the experiment, with completion rewarded by another 15€/CHF gift card. 18 children (9 females) completed the second phase, with the following age distribution: 5 years: *n* = 2; 6 years: *n* = 3; 7 years: *n* = 1; 8 years: *n* = 4; 9 years: *N* = 6; 10 years: *N* = 3.

### 2.2 Data collection

#### 2.2.1 Adults

On the website, participants were informed that they were invited to take part in a 14-day experiment, with each daily session taking approximately 2 min. The experiment consisted of a series of bets, each offering the chance to win a specific number of points, and participants were encouraged to maximize their total score. A virtual podium displayed a leaderboard showing each participant’s total points and rank. To participate, subjects had to create an account by providing a nickname (used on the leaderboard), an email address and a password, as along with basic demographic information, such as age, sex and occupation. No personal identifiers, such as full names were collected. The first phase of the experiment lasted 10 consecutive days, with participants required to log in daily, while the second phase spanned 4 consecutive days. Daily reminder emails were sent to encourage consistent participation. The website was specifically developed for this experiment and hosted by WebFreeHosting.net. Data were stored on a MySQL server, accessible only to the researchers.

#### 2.2.2 Children

On the website, participants were invited to take part in a 22-day experiment, with each daily session lasting approximately 5 min. They learned that the experiment consisted of a series of mathematical questions, each offering the chance to win a certain number of marbles. The homepage displayed the list of all participants, along with a countdown of the remaining days before they could claim their reward. To participate, parents were required to create an account for their child by providing a nickname (used on the homepage), an email address, the child’s age, and sex. No personal identifiers such as the child’s full name were collected. The first phase of the experiment lasted 16 consecutive days, with children logging in daily, while the second phase spanned 6 consecutive days. Daily reminder emails were sent to parents to encourage participation. The website was developed specifically for this experiment and hosted on WebFreeHosting.net, with the data stored on a MySQL server, accessible only to the researchers. Upon completion of the experiment, parents received an email requesting that they sign a parental agreement for the use of their child’s data, after which the chosen gift card was sent.

### 2.3 Experimental design

#### 2.3.1 Study 1

Participants completed two experiments, Experiment 1.1 (single-cup design; duration of 6 days for adults, 10 for children) and Experiment 1.2 (multi-cup design; duration of 4 days for adults, 6 for children). After creating their account, the order in which they performed Experiments 1.1 or 1.2 was randomly assigned. At the beginning of each experiment, a brief message outlined its specific rules.

##### 2.3.1.1 Experiment 1.1

In this experiment, participants were asked to choose between a safe option (Option A, which always yielded 2 points) and a risky option (Option B, which yielded an unknown number of points). For adults, the instructions read as follows: “The goal of this experiment is to win a maximum number of points. During each trial, you’ll choose between Option A, which will give you 2 points, and Option B, which gives you an unknown number of points. After each trial, the number of points for option B will be revealed.” and for children “I will show you where I hide my marbles. Under the red cup, I always hide two marbles. Under the blue cup, I can’t remember how many marbles I hid! You can have my marbles: if you pick one of the cups, you will win all the marbles hidden under it.”

Subjects were not informed of the probability (P) of winning Option B or of the number of points (N) associated with it. Depending on these values, the expected gain (EV = P × N) of Option B was either higher or lower than the 2 points guaranteed by Option A, rendering the gamble advantageous or disadvantageous. The various P-N combinations and their corresponding expected gains (EV = P × N) are shown in Table 1 (each combination in Table 1 is assigned to a specific day of the experiment and the order of combination presentation was randomized between participants, see Supplementary Figure 1 for a diagram of Experiment 1.1).

**TABLE 1:** Expected value (EV) of the risky Option B as a function of the win probability (P) and reward value (N) in Experiments 1.1 and 2.1.

Value N	Probability P			
Value P	0.25	0.33	0.55	1
2	–	–	1	–
4	1	1.33	2	4
6	–	–	3	

Conditions with *P* = 0.33 were not tested with children. Each condition (table cell) represents 40 trials per subject (i.e., 1 day of testing for adults and 2 days for children) in Experiment 1.1, and 10 trials per subject in Experiment 2.1.

Each daily session consisted of 40 trials for adults and 20 trials for children, divided in 4 or 2 blocks of 10 trials, respectively. In each trial, participants could win a certain number of points (marbles for children), and a counter on the website displayed the points (marbles) earned that day, updating after each bet. At the end of each session, participants were reminded of the points they earned that day, along with their updated total score and rank.

##### 2.3.1.2 Experiment 1.2

Participants chose between a safe option (Option A, which always yielded 2 points) and a risky option (Option B) that was divided into several sub-options, awarding either 0 points or a given number of points (with only one of the sub-options awarding points). For adults, the instructions read: “The goal of this experiment is to win as many points as possible. In each trial, you will choose between Option A, which gives you 2 points, and Option B. Option B is divided into several sub-options, but only one sub-options will yield points while the others give 0. The number of points for the winning sub-option is shown at the top of the page. You must choose between Option A and one of the sub-options for Option B. After each trial, the winning sub-option will be revealed.” and for children: “I will show you where I hide my marbles. Under the red cup, I always hide two marbles. The other 6 marbles are hidden under one of the green cups, but I can’t remember which one! If you pick a cup, you will win all the marbles hidden under it. The picture shows you how many green cups there are and the number of marbles hidden under one of them.

Subjects were provided with explicit written information about reward value (N) available from one of the sub-options of Option B, and could figure out the probability of winning the reward by the number of those sub-options (*P* = 1/number of sub-options). Depending on these values, the expected gain (P × N) of a sub-option of Option B was either higher or lower than the 2 points offered by Option A, making the gamble advantageous or disadvantageous. The 16 different values for the combination of P and N, along with their associated expected gains, are shown in Table 2 (see Supplementary Figure 1 for a diagram of Experiment 1.2).

**TABLE 2:** Expected value (EV) of the risky Option B as a function of the win probability (P) and reward value (N) in Experiments 1.2 and 2.2.

Value N	Probability P			
Value P	0.25	0.33	0.55	1
2	0.5	0.66	1	2
4	1	1.33	2	4
6	1.5	2	3	6
7	1.75	2.33	3.5	7

Conditions with *N* = 7 was not tested with children. P is determined by the number of sub-options: 0.25 if there are 4 sub-options, 0.33 if there are 3 sub-options, 0.5 if there are 2 sub-options, and 1 if there is 1 sub-option. Each condition (table cell) represented 10 trials per subject (adults and children) in Experiment 1.2 and 5 trials per subjects in Experiment 2.2.

For adults, Option B could yield *N* = 2, 4, 6, or 7 points. Each day, participants completed one block of 10 trials for each reward level, and those four daily blocks were presented in a randomized order. For each trial within a block, the number of sub-options of Option B was randomly selected from 1 to 4, yielding a win probability of 1 (if 1 sub-option), 0.5 (if 2 sub-options), 0.33 (if 3 sub-options) or 0.25 (if 4 sub-options). The duration of the experiment was 4 days, so each reward level (i.e., each line in Table 2) was tested 40 times, and each condition (i.e., each in Table 2 cell) was tested 10 times in Experiment 1.2.

For children, Option B could yield *N* = 2, 4, or 6 points. As for adults, four blocks of 10 trials were administered for each reward level, and for each trial within a block, the number of Option B’s sub-options was randomly selected from 1 to 4. Children performed a total of 2 blocks (20 trials) per days, with the 12 blocks also presented in a randomized order.

#### 2.3.2 Study 2

Six months after completing the first phase, participants were invited to take part in modified replications of the original tasks, referred to as Experiment 2.1 (modified version of Experiment 1.1) and Experiment 2.2 (modified version of Experiment 1.2). The order in which participants completed Experiments 2.1 and 2.2 was randomly assigned. The overall experimental design was comparable to that used in Study 1: for adults, each daily session comprised 40 trials divided into 4 blocks of 10 trials, while for children, each session consisted of 20 trials divided into 2 blocks of 10 trials. In every trial, participants could win a specific number of points, with a counter on the website displaying the points earned during the day that updated after each bet. At the end of each session, participants were reminded of the points earned that day. For adults, their updated total score and rank were also provided.

##### 2.3.2.1 Experiment 2.1

This was a replication of Experiment 1.1, with one key modification: participants were explicitly informed (by written instructions) of both the reward value and the probability associated with the risky option. In Experiment 2.1, participants chose between a safe option (Option A, which always yielded 2 points) and a risky option (Option B), for which the reward and its probability were stated by written description. For example, the risky option was described as: “Option B: 1 chance out of 4 of winning 2 points; otherwise, you win 0 points”. In the children version, the written description was complemented by a visual display on the risky cup, showing both the number of marbles to be won and the probability of winning them. The P – N combinations were identical to those in Experiment 1.1 (see Table 1 and Supplementary Figure 2 for a diagram of the Experiment 2.1). However, each combination was tested over 10 consecutive trials rather than 40. This modification ensured that participants relied on the written probability descriptions rather than adjusted their perceptions based on trial-by-trial feedback.

##### 2.3.2.2 Experiment 2.2

This was a replication of Experiment 1.2, with one key difference: instead of inferring the win probability from the number of risky sub-options, participants received this information through written instructions. Subjects had to choose between a safe option and a single risky option of known reward value and probability to win. The written instructions were similar to those in Experiment 2.1: “Option B: 1 chance out of 4 of winning 2 points; otherwise, you win 0 points.” For children, a visual representation on the risky cup displayed the number of marbles to be won and the associated probability. The P – N combinations were identical to those in Experiment 1.2 (see Table 2 and Supplementary Figure 2 for a diagram of the Experiment 2.2), but each combination was tested over 5 trials instead of 10.

### 2.4 Ethics assessment

The research protocol was assessed and approved by the University of Neuchatel’s Ethics Commission (Permit number 73/2020). Parental informed consent was obtained for all infant participants, in accordance with ethical guidelines and legal requirements.

### 2.5 Statistical analysis

For the analysis, we fitted a generalized linear mixed model with binomial error structure and logit link function to our data on subjects’ choices. The response variable was subjects’ choice, i.e., the safe or risky option. The data were analyzed using the glmer function of the lme4 package (version 1.1-36) in R, using R version 4.4.3. We checked the normality and the homoscedasticity of plotted residuals and their independence with respect to fitted and other predictors to ensure we met the assumptions of the model. The significance of each predictor variable in explaining variation in rate of risky choices was tested by an analysis of deviance (type II Wald chi-square test).

When comparing risk-preference data in Experiments 1.1 and 1.2, we fitted the value and the probability of the risky option as continuous variables and experimental design, sex and age as a categorical variable (adult or children). We also fitted the two-way interactions between economic parameters (probability and value of the risky option) and experimental design and age, the two-way interaction between age and protocol. We fitted random intercept for subjects and random slopes of experimental design within subjects and blocks. We compared this full model to a null model (no fixed effects and the same random structure as the full model) using a Likelihood Ratio Test.

If the comparison showed a significant difference, we assessed the significance of each predictor variable in explaining variation in rate of risky choices by an analysis of deviance (type II Wald chi-square test). Then, starting with the highest-level interaction terms, we removed non-significant terms one by one. The final model where all non-significant interaction terms were removed is presented in the result section.

A post-hoc power analysis was conducted using the powerSim function of the simr package (version 1.0.7). Power was estimated for each fixed predictor by simulating 100 datasets, at α = 0.05. A sensitivity analysis was also conducted by simulating 100 datasets across a range of effect sizes to determine, for each predictor, the smallest effect size detectable with a 80% power at α = 0.05.

Finally, on the final model, we ran post-hoc tests to calculate estimated marginal means or estimated trends (functions emtrends or emmeans of the emmeans package, version 1.10.7).

Estimated marginal means refer to the mean response values for each group/condition, adjusted for the effects of other variables in our model. Estimated marginal means offer an interpretation of group differences that accounts for covariates, providing a clear comparison across groups (for instance, comparison of the level of risky choice for each age group at the indifference point, for each experimental design).

In particular, in order to check whether the level of risky choice for EV = 2 was significantly different from 50%, we calculated the estimated marginal means for EV = 2 (equivalence point). If the level of risky choice was not significantly different from 50%, that would indicate risk-neutrality (subjects have no preference between a safe and a risky option of equal expected values). On the contrary, a level of risky choice for EV = 2 significantly higher than 50% would indicate risk-proneness (subjects prefer the risky option), and a level of risky choice significantly lower than 50% would indicate risk-aversion (subjects prefer the safe option).

To assess how changes in the experimental design influenced risk-preferences, we combined the data from all four Experiments (1.1, 1.2, 2.1, 2.2) using only the subjects that completed all experiments. We first evaluated the overall levels of risky choices by calculating the estimated marginal means from the generalized linear mixed model. Next, we analyzed the sensitivity to the economic parameters (i.e., reward value and win probability) by estimating the corresponding trends. Those trends indicate patterns or directions of change observed in the estimated marginal means across conditions or time points.

This integrative approach provided clear insights into how the design types and the applied modifications affected both the baseline risk preference and the responsiveness to specific economic cues.

## 3 Results

### 3.1 Study 1

In Experiment 1.1, the “single-cup” design, participants chose between a safe option (always yielding 2 points) and a risky option (yielding a variable number of points). In Experiment 1.2, the “multi-cup” design, the safe option was identical, but the risky option was a tray of risky sub-options, only one of which yielded a variable number of points.

We fitted a generalized liner mixed model incorporating all predictors (economic parameters, experimental design, participants age group and sex) and their interactions, which differed significantly from the null model (LRT: *χ*^2^(10) = 12325, *p* < 0.001; Supplementary Tables 1–3). Power analysis showed adequate power (>80%) for all significant predictors, and the sensitivity analysis showed the study was sufficiently powered to detect small to medium effects for primary predictors (see Supplementary Table 4 for power levels for each predictors and minimal detectable effect size for each predictors).

**FIGURE 1:**
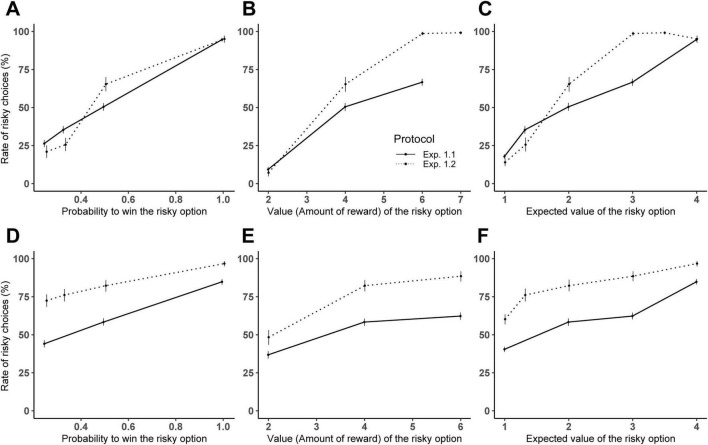
Comparison between Experiments 1.1 and 1.2 in adults **(A–C)** and children **(D–F)**. Mean percentage of trials where subjects selected the risky option for Experiment 1.1 (solid line) and Experiment 1.2 (dotted line): **(A,D)** according to the value of the risky option (only trials with *P* = 0.5 are considered to allow experiment comparison); **(B,E)** the probability to win (only trials with *N* = 4 are considered); **(C,F)** the expected value (only trials with *P* = 0.5 or *N* = 4 are considered). Error bars indicate 95% confidence intervals.

In both experiments, subjects increasingly chose the risky option both as the probability of winning increased (*χ*^2^(1) = 3781.02, *p* < 0.001) and as the value of the risky option increased (*χ*^2^(1) = 2796.14, *p* < 0.001). The experimental design (*χ*^2^(1) = 16.27, *p* < 0.001) significantly influenced their risk-taking behavior (see Figure 1) and the age group of subjects (*χ*^2^(1) = 3.07, *p* = 0.07) marginally influenced risk-taking behavior. There was a significant interaction between the experimental design and the value of the risky option (χ2(1) = 447.61, *p* < 0.001), and between the experimental design and the probability to win (χ2(1) = 140.15, *p* < 0.001), as well as between the age group and the value of the risky option (χ2(1) = 316.79, *p* < 0.001), between the age group and the probability to win (χ2(1) = 316.80, *p* < 0.001) and between the age group and the protocol (χ2(1) = 19.56, *p* < 0.001).

#### 3.1.1 Indifference point

Analysis at the indifference point (i.e., where both the safe and the risky options have an expected value of 2) revealed differences between the two experiments: in Experiment 1.1, the percentage of risky choices did not differ significantly from 50% (estimated marginal means: [0.47; 0.57] for adults and [0.48; 0.63] for children), indicating risk-neutral behavior. In contrast, Experiment 1.2 showed a significant increase in risky choices at the indifference point for both adults and children (estimated marginal means: [0.62; 0.81] for adults and [0.92; 0.98] for children), demonstrating risk-prone behavior.

Our result thus showed a risk-preference shift between the single-cup design (where subjects where risk-neutral) and the multi-cup design (where subjects were risk-prone), especially in children.

#### 3.1.2 Sensitivity to economic parameters

We conducted post-hoc tests assessing subjects’ sensitivity to the economic parameters across both experimental designs. The trend estimates for the value of the risky option were significantly steeper in Experiment 1.2 (adults: 1.83, SE = 0.05: children: 1.39, SE = 0.06) than in Experiment 1.1 (adults: 0.74, SE = 0.01; children: 0.30, SE = 0.03). Similarly, the trend estimates for the probability to win were steeper in Experiment 1.2 (adults: 8.19, SE = 0.28; children: 6.50, SE = 0.31) compared to Experiment 1.1 (adults: 4.69, SE = 0.09, children: 3.00, SE = 0.12).

These results highlight that the multi-cup design (Experiment 1.2) not only promoted risk-prone behavior but also heightened sensitivity to both reward value and probability, with adults showing a greater sensitivity than children across both experiments.

### 3.2 Study 2

We then conducted two follow-up experiments, analogous to the previous ones, to investigate the mechanisms underlying the risk-preference shift we described. In Experiment 2.1 (a modified version of Experiment 1.1) subjects were informed about both the reward value and the success probability of the risky option by written instructions. Thus, Experiment 2.1 is a description-based version of Experiment 1.1 (which is originally an experience-based experiment).

In Experiment 2.2 (a modified version of Experiment 1.2), instead of choosing between one safe option and several risky sub-options, subjects had to choose between one safe option and one risky option of known reward value and probability (thanks to written instructions). Thus, Experiment 2.2 is a low-exploration version of Experiment 1.2 (where the risky option, composed of different sub-options, could trigger high levels of explorations).

The key difference between Experiments 2.1 and 2.2 is the order of trial presentation. In both experiments, the overall reward value of the risky options remained constant over all trials conducted within a block, but probability of winning could vary from trial to trial (Experiment 2.2 only).

**FIGURE 2:**
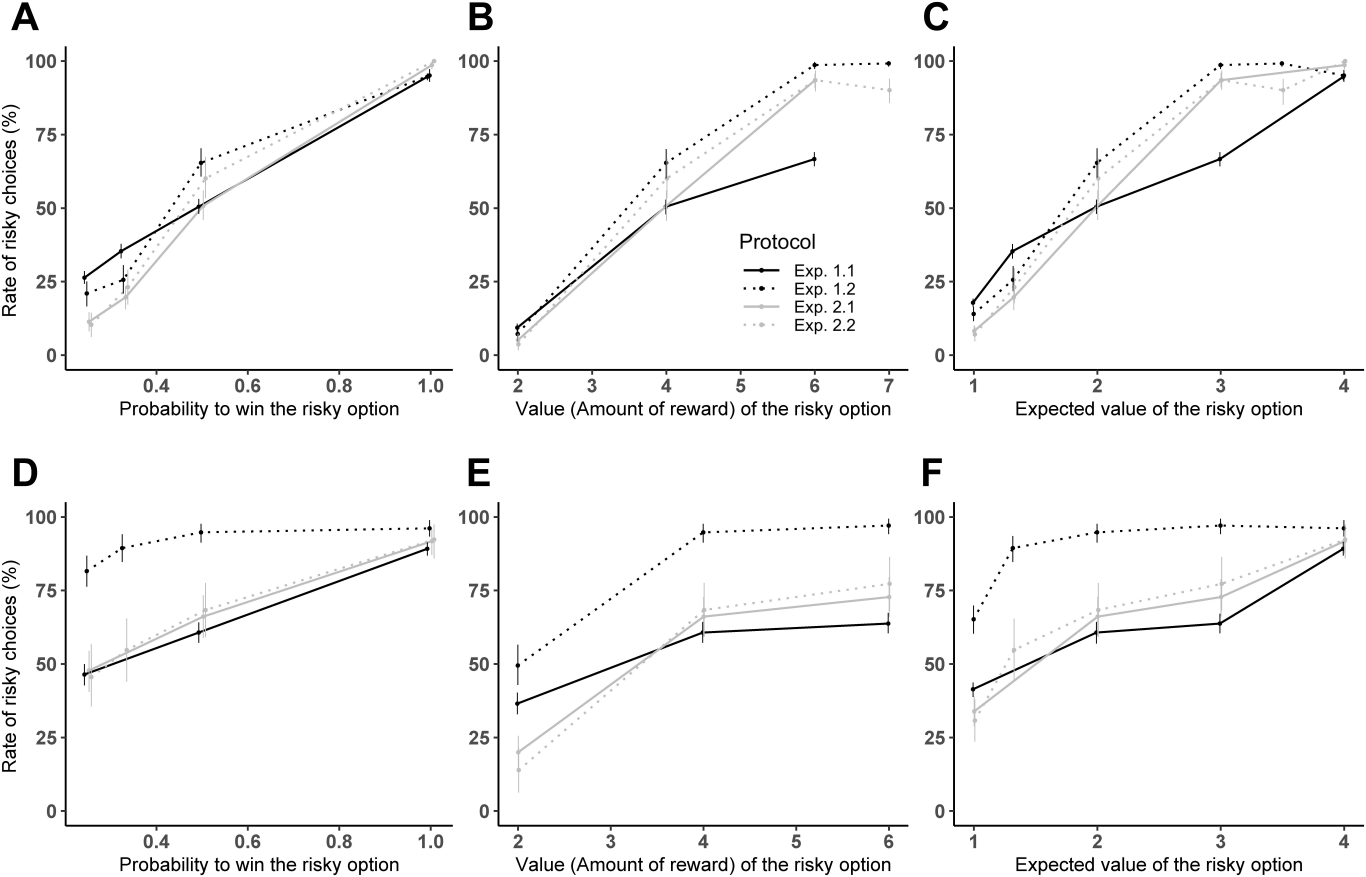
Performance comparison between study 1 and study 2 in adults **(A–C)** and children **(D–F)**. Mean percentage of trials where subjects selected the risky option for the two experiments of study 1: Experiment 1.1 (solid black line), and 1.2 (dotted black line), and the two experiments of study 2: Experiment 2.1 (solid gray line) and 2.2 (dotted gray line). The percentage of risky choices is showed: **(A–C)** according to the value of the risky option (only trials with *P* = 0.5 are considered to allow experiment comparison); **(B–E)** according to the probability to win (only trials with *N* = 4 are considered); **(C–F)** according to the expected value (only trials with *P* = 0.5 or *N* = 4 are considered). Error bars indicate 95% confidence intervals.

To evaluate the impact of these design modifications on subjects’ choices, we combined the data of both studies (see Figure 2) and fitted a full model using the same random structure as the one we used before (Experiments 1.1 and 1.2). This full model, which included all predictors variables and interactions, was significantly different from the null model (LRT: *χ*^2^(17) = 9137, *p* < 0.001; Supplementary Tables 5–7). Power analysis showed adequate power (>80%) for all significant predictors, and the sensitivity analysis showed the study was sufficiently powered to detect small to large effects for primary predictors (see Supplementary Table 8 for power levels for each predictors and minimal detectable effect size for each predictors).

#### 3.2.1 Indifference point

Estimating the marginal means at the indifference point showed that subjects maintained risk-neutral behavior in Experiment 2.1 (EMM interval: adults [0.36; 0.56], children: [0.48; 0.75]), mirroring results from Experiment 1.1 (EMM interval: adults: [0.42; 0.52], children: [0.50; 0.65]). This result shows that switching from experience-based knowledge of economic parameters (Experiment 1.1, where subjects have to use repeated feedback of the risky option to evaluate the reward and probability to win) to description-based knowledge of those parameters (Experiment 2.1) did not alter risk-preference in either age group.

In contrast, switching from a tray of risky options (Experiment 1.2) to a single risky option of known economic parameters (Experiment 2.2), led to a notable shift in risk-taking. Adults transitioned from risk-proneness in Experiment 1.2 (EMM interval: [0.46; 0.79]) to risk-neutrality in Experiment 2.2 (EMM interval: [0.39; 0.60]). Although children remained risk-prone in Experiment 2.2, their levels of risky choices were significantly reduced compared to Experiment 1.2 (EMM interval decreased from [0.96; 1] to [0.53; 0.79]).

#### 3.2.2 Sensitivity to economic parameters

We then compared the estimated trends for the two key economic parameters, probability to win and reward value, across experimental conditions (Supplementary Table 9). For success probability, we found that the trend estimate was flatter in Experiment 1.1 (adults: 5.55, SE = 0.15; children: 2.63, SE = 0.16) compared to the other three experimental conditions (trend estimate for adults: 8.95, SE:0.49 in Experiment 1.2; 8.27, SE:0.35 in Experiment 2.1 and 8.51, SE:0.49 in Experiment 2.2 and trend estimate for children: 6.02, SE:0.51 in Experiment 1.2; 5.35, SE:0.35 in Experiment 2.1 and 5.58, SE:0.51 in Experiment 2.2).

Thus, the sensitivity to the probability to win was lowest in Experiment 1.1, i.e., the experiment in which subjects had to estimate probability by experience and not by reasoning (as in Experiment 1.2) or by written description (as in Experiments 2.1 and 2.2).

For reward value, the trend estimate was also flat in Experiment 1.1 (adults: 0.77, SE = 0.01; children: 2.63, SE = 0.16) but became steeper in Experiment 1.2 (adults: 2.45, SE = 0.10; children: 6.02, SE = 0.52). In Experiments 2.1 and 2.2, the trends estimate for reward value were intermediate, with adults showing estimates of 1.43 (SE = 0.05) in Experiment 2.1 and 1.39 (SE = 0.07) in Experiment 2.2, and children showing estimates of 5.35 (SE = 0.35) in Experiment 2.1 and 5.58 (SE = 0.51) in Experiment 2.2.

These results indicate that the sensitivity to the reward value was also the lowest in Experiment 1.1, the only one where subjects had to estimate it by experience. It also indicates that the sensitivity to the reward value was higher in Experiment 1.2 (multiple-cup design) than in Experiments 2.1 and 2.2, even though the reward value was indicated by written instructions similarly in all three designs, showing that the multiple-cup designs enhanced the saliency of the economic parameters of the risky option compared to the other designs.

## 4 Discussion

In this study, we first replicated, with human subjects of two age groups (adults and children), an experimental design (Study 1) that was previously used on orang-utans and gorillas (Lacombe et al., 2022) and had shown a reversal of behavior between two risk-assessment tasks. In this experiment, a “single-cup” design (Experiment 1.1) led to risk-neutrality or risk-aversion in ape subjects, and a “multi-cup” design (Experiment 1.2) led to risk-proneness in apes, although both designs had exactly the same payoffs.

Our replication of this study showed that both adults and children exhibited a similar shift in risk preference depending on the experimental design: subjects were risk-neutral in the single-cup condition and risk-prone in the multi-cup condition. Notably, this preference shift was more pronounced in children, who displayed particularly high risk proneness in the multi-cup design.

This aligns with previous work that showed consistent risk-proneness in children compared to adults (Levin and Hart, 2003; Levin et al., 2007), as risk-aversion seems to increase with age (Harbaugh et al., 2002; Rakow and Rahim, 2010; Broihanne and Dufour, 2018), though this progression is variable and context-dependent (Slovic, 1966; Broihanne and Dufour, 2018), influenced by factors such as cognitive ability, personality, rationality, and impulsivity (Levin et al., 2007).

In addition, our study showed that children’s risk-proneness was increased in the “multi-cup” design. This lines up with previous work (Rakow and Rahim, 2010; Van Duijvenvoorde et al., 2012; Rolison et al., 2022) which showed that the level of risk-proneness in children depends on which task is used, as each economic task can demand specific cognitive abilities in which children are at different developmental stages.

This risk-preference shift could be due to the description-experience gap, that has been documented in children (Rakow and Rahim, 2010), adults (Hertwig and Erev, 2009), and non-human primates (Heilbronner and Hayden, 2016). This gap was initially considered to only induce probability distortion (Hertwig et al., 2004), but was more recently shown to also interact with risk-assessment behaviors and affecting for instance framing effect (Ludvig and Spetch, 2011). Consequently, the description-experience gap could be a candidate hypothesis explaining the risk-preference shift that was described in our study in humans of different age group or in Lacombe et al. (2022) in gorillas and orang-utans.

While the impact of the description-experience gap in children choice has been already investigated (Rakow and Rahim, 2010; Rolison et al., 2022), it is difficult to compare two tasks that are very different (and not only in how probabilities are presented, i.e., by computing feedbacks in experience-based design or by instructions in description-based designs).

For instance, in Rolison et al. (2022), the experience-based task that was used is the “Hungry Squirrel task” where a squirrel can cross a bridge to collect acorns (the longer he goes, the more acorns he can collect), while risking the bridge to be broken at each steps the squirrel takes. The number of steps children choose to take before collecting the acorns and returning to the banks indicates their levels of risk-proneness. Also in Rolison et al. (2022), the description-based task that was used is the “Pirate task”, a lottery task where children have to choose a chest belonging to one of two pirates, a “safe pirate” owning chests of identical and known amount of coins, and a “risky pirate” owning several chests, only one of which containing a certain a known amount of coins.

The difference between these two tasks does not allow for direct comparison, as several aspects of the tasks are different (squirrel versus smiling pirate, collecting acorns instead of coins,…).

In our study, we compare two tasks that are similar in all aspects but how variance is presented, which allows for direct performance-comparison between an experience-based experiment (Experiment 1.1, where subject have to use the feedback of each trial to evaluate the payoff of the risky option) and a non-verbal (thus, usable for apes or young children) description-based experiment (Experiment 1.2, where the probability to win the risky option is deducible from the number of cups, without prior experience).

However, in Study 2, we replicated a modified version of the single-cup design by adding written information about the risky payoffs, thus making it a description-based task instead of an experience-based task. This modification did not alter risk-preference and subjects of all age group remained less risk-prone than in the multi-cup design. It is thus unlikely that the risk-preference shift described between the single-cup and the multi-cup in Study 1 is due to the experience-description gap, and we must reject that hypothesis.

Another possible hypothesis is that the risk option is more salient in multi-cup design than in the single-cup design, thus triggering more curiosity-oriented or more exploration behavior, especially in children and apes. Indeed, children decision-making is more triggered by curiosity (Liquin and Lombrozo, 2020) and a willingness to explore (Liquin and Gopnik, 2022; Roig et al., 2025) than adults’.

First, we can reject the hypothesis that the higher levels of risk-proneness observed in the multi-cup design was due to the fact that this design triggers more exploration strategies than exploitation strategies [in the classical trade-off between exploitation and exploration in decision making (Cohen et al., 2007)]. Indeed, both designs provide full feedback after each trial (the content of every cup is shown after each trial, whatever cup the subject chose), thereby reducing the incentive to explore the risky option to gain information.

However, we can make the hypothesis that the multi-cup design triggered a preference for the risky option as it is more salient in this design (bigger, larger, and with an animation showing the possible payoff in the children task), than in the single-cup design, which is likely to focus attention and thus impact decision-making. Indeed, Orquin et al. (2018) showed that the surface size, the set size, and the salience of a stimulus can attract attention, and Orquin and Mueller Loose (2013) showed that salient objects are thus more likely to be chosen by a decision-maker. Others findings (Kwak and Huettel, 2018) suggest that decision in risky tasks is linked to which option is presented first or perceived first, although the impact on decision-making of which option is seen first is still debated (Van Der Laan et al., 2015). More recently, Blanco and Sloutsky (2020) showed that in a simple decision task, manipulating the saliency of options lowered children preference for higher-payoffs options, while this manipulation had low impact on decision-making in adults.

We can thus make the hypothesis that, in the multi-cup design, the risky option was more salient than the safe option (and more salient than the risky option in the single-cup design), thus drawing subjects’ attention and influencing their preference for that option. Indeed, in Study 2, our replicate of the multi-cup design in which the risky option was replaced by a single option of known payoff showed lower risk-proneness in children and risk-neutrality in adults.

Finally, a last hypothesis to explain the preference-shift between the single-cup and the multi-cup design is that the two designs induce different framing of the safe and the risky options, resulting in risk-neutral behavior in the single-cup design and risk-prone behavior in the multi-cup design.

According to Prospect Theory, when faced with a choice between two options, subjects evaluate these options relative to a reference point (the *status quo*). Options perceived as smaller than this reference are considered as losses, while those larger are viewed as gains. A majority of studies showed that decision-makers [humans of all age groups (Tversky and Kahneman, 1991; Schlottmann and Tring, 2005), primates (Krupenye et al., 2015), others animals (Marsh and Kacelnik, 2002),…] are risk-averse if the choice is framed positively (gain context), and risk-prone if the choice is framed negatively (loss context), independently of the actual quantitative values of all options.

In our study, we make the hypothesis that subjects use different stimuli of the task to define their reference point (or *status quo*, need, or desire). In the single-cup design, they use the value of the safe option as their reference point (as it is the only payoff they have explicit information on), and in the multi-cup design, they use the value of the risky option as their reference point (the higher of the two known payoffs).

Thus, choosing the safe option in the single-cup design secures the *status quo*, while selecting the risky option offers a chance to earn more than the *status quo* or nothing at all. This setup corresponds to a risk-assessment task in the gain domain, where humans typically exhibit risk-averse behavior – which is consistent with our findings.

In contrast, in the multi-cup design, choosing the safe option results in a loss relative to the *status quo*, while selecting the risky option can lead to either a greater loss (if an unbaited sub-option is chosen) or no loss (if the baited sub-option is selected). This setup corresponds to a risk-assessment task in the loss domain, where humans tend to be risk-prone – which is consistent with our results.

The observation that subjects exhibited greater sensitivity to the value of the risky option in the original multi-cup design – despite receiving comparable explicit written information about the risky option’s value in our replication of the multi-cup design in Study 2 - further supports this hypothesis.

The mechanism behind the choice of the safe option as the reference point in the single-cup design and of the risky option in the multi-cup design could also be due to the difference in salience of both options in the two designs, as discussed before. When a certain option is available in a decision-making task, it is often used as a reference point (Rakow and Rahim, 2010), but this could be altered by the high salience of the risky option in the multi-cup design.

Future work is needed to explore the candidate mechanisms explaining the contextual preference-shift we described. First, in order to investigate the impact of attention on risk-preference shift between the single-cup and the multi-cup design, it would be useful to replicate our study using an eye-tracker to control for attention, or manipulate the saliency of each option. Additionally, in order to investigate if differences in framing could account for the preference-shift described between the two designs, a same reference point could be imposed for both designs (single-cup and multi-cup design), for instance using a method similar to Wyszynski and Diederich (2022) (where subjects have a certain amount of points at the beginning of each experiment, and before each trial are told how many points are removed from the budget -thus setting a reference point- and are then offered to bet or not to keep a certain portion from those points).

Finally, it would be important to confirm and precise our findings with a larger dataset for the children group, and to increase the sample size for each age class in this group. Indeed, given the duration of our experiment (22 days) imposed by the choice of within-subjects designs, our sample size was reduced and smaller than was targeted according to Defoe et al. (2015), thus preventing to conduct a comparison of performance for each age class in the children group. Future studies should replicate this protocol with larger sample size of each age group to study more precisely the development of behavior during childhood. For a more precise comparison of performance across age group, it would also be important to replicate the experiment using the same incentives for adults and children.

It could also be important to add attention checks before each trial could also precise and confirm our findings, as no attention checks were conducted in our study.

Our results provide strong evidence for evolutionary conservation of the cognitive processes underlying risk-taking behavior. The patterns of risk assessment observed in this study closely mirror those found in our companion study with non-human primates. This supports the view that many neural and cognitive mechanisms governing decision-making have been preserved throughout primate evolution (Rangel et al., 2008). Several of these mechanisms likely originate from fundamental problem-solving skills—such as foraging, sensitivity to salient cues, and context-dependent evaluation of uncertainty—that have clear adaptive value for survival in unpredictable environments. Notably, contextual framing and a propensity for exploration appear to be deeply rooted, evolutionarily adaptive strategies that continue to shape decision-making in modern humans.

## 5 Conclusion

Our findings demonstrated that context plays a crucial role in risk-related decision-making, in both adult and child participants, as well as in non-human primates (Lacombe et al., 2022). Both human and non-human primates showed similar patterns of seemingly irrational behavior, tending toward risk-prone or risk-averse strategies depending on how options were presented. A key factor was whether the risky choice appeared as a single option (single-cup design) or as part of a tray with multiple options (multi-cup design), with the latter typically eliciting more risk-prone behavior. By making subtle modifications to these designs, we were able to identify cognitive mechanisms likely driving risk-taking, as a general tendency for exploration and contextual framing, emerging as particularly promising explanations in both humans and animals.

## Data Availability

The datasets presented in this study can be found in online repositories. The names of the repository/repositories and accession number(s) can be found below: doi: 10.5281/zenodo.15412216.

## Footnotes

1
https://www.zoobasel.ch/en/freundeverein/r/53/zolligumper/
