# Supplementary material for: Contextual influences on risk-taking in children and adults
Source: Front Behav Neurosci. 2025 Oct 22;19:1644777. doi: 10.3389/fnbeh.2025.1644777 (PMC12586073; doi:10.3389/fnbeh.2025.1644777)
Supplement: Supplementary file 1 [file Data_Sheet_1.pdf]

frontiers
frontiers

Supplementary Material

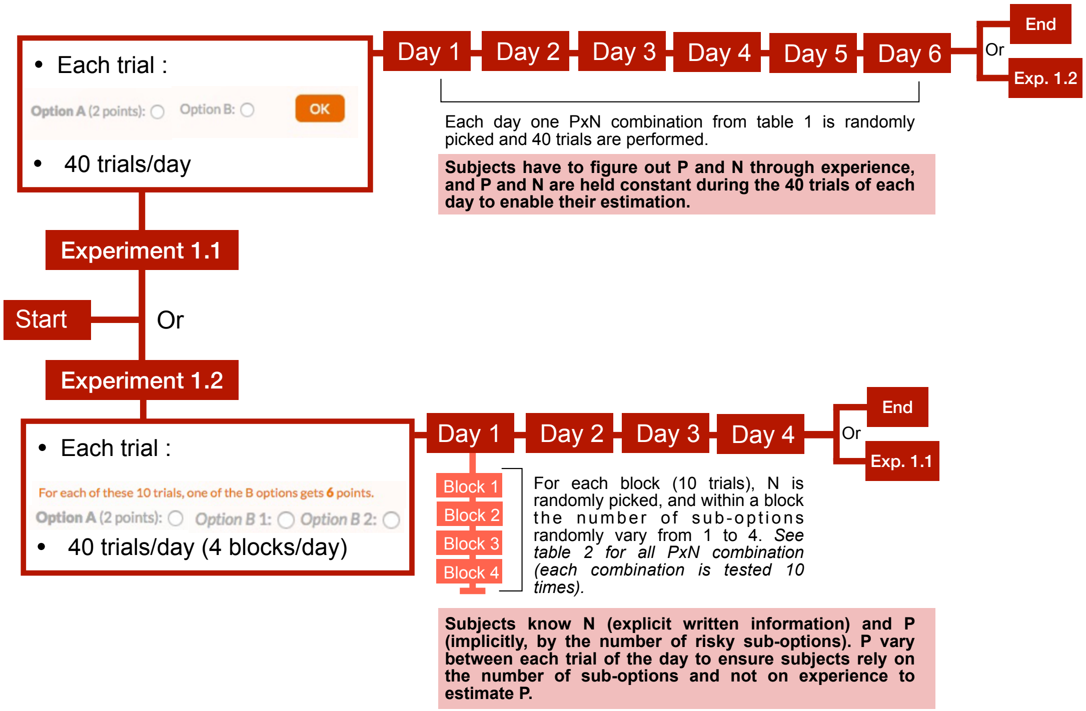

*

Supplementary Figure 1: Experimental design of study 1 for adults. For children, in Experiment 1.1, one PxN combination was not tested and the number of daily trials was reduced to 20, so the experiment took 10 days to be completed rather than 6. For children in Experiment 1.2, one value of N was not tested and the number of daily trials was again reduced to 20, so the experiment took 6 days to be completed rather than 4.

Supplementary Material

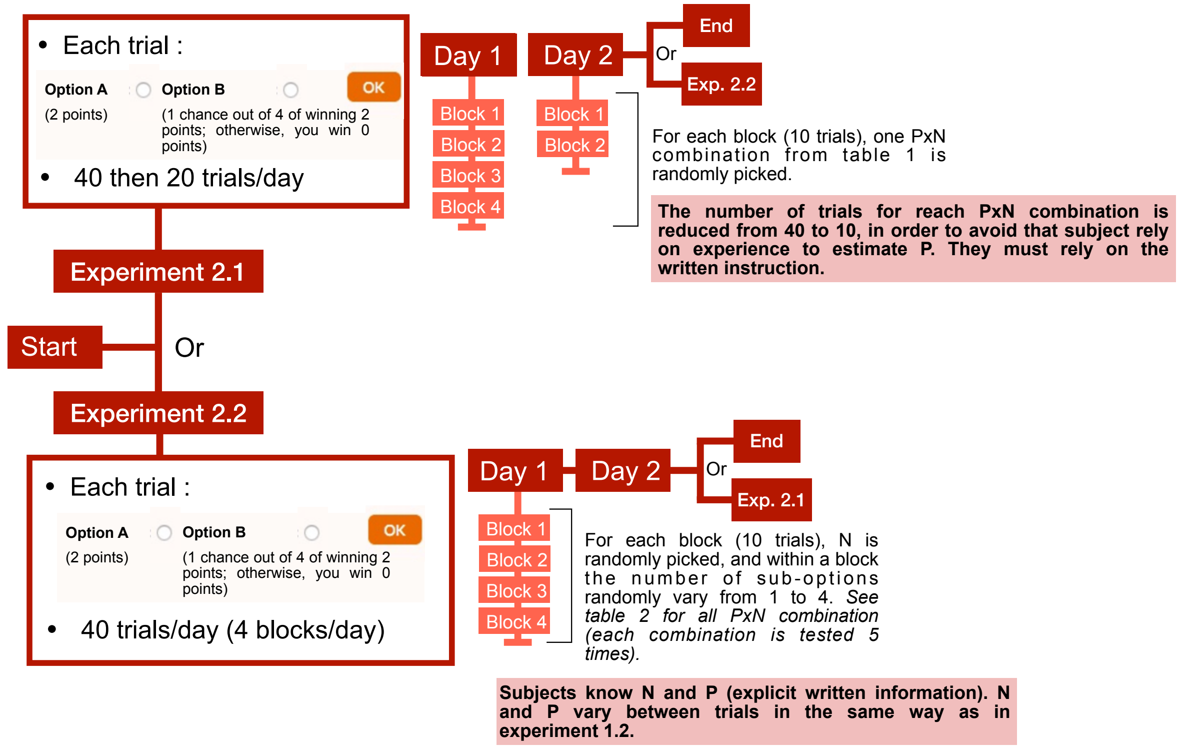

Supplementary Figure 2: Experimental design for Study 2 in adults. For children, in Experiment 1.1, one PxN combination was not tested and the number of daily trials was reduced to 20, so the experiment took 3 days to be completed rather than 2. For children in Experiment 1.2, one value of N was not tested and the number of daily trials was again reduced to 20, so the experiment took 3 days to be completed rather than 2.

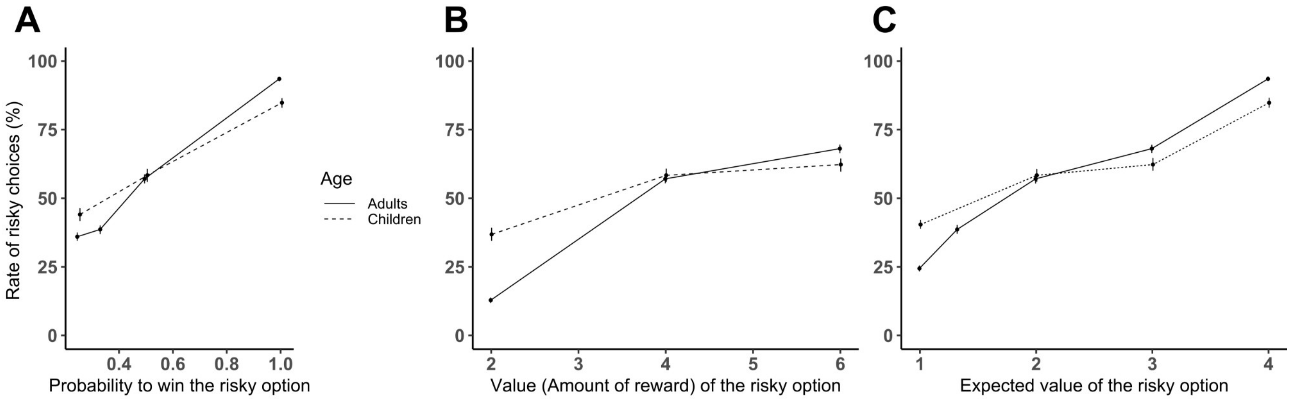

Supplementary Figure 3: Performance comparison between adult and children in Experiment 1.1. Mean percentage of trials where subjects selected the risky option for Experiment 1.1 between adults (solid line) and children (dotted line): (A) according to the value of the risky option (only trials with P=0.5 are considered to allow experiment comparison) and (B) the probability to win (only trials with N=4 are considered) and (C) the expected value (only trials with P=0.5 or N=4 are considered). Error bars indicate 95% confidence intervals.

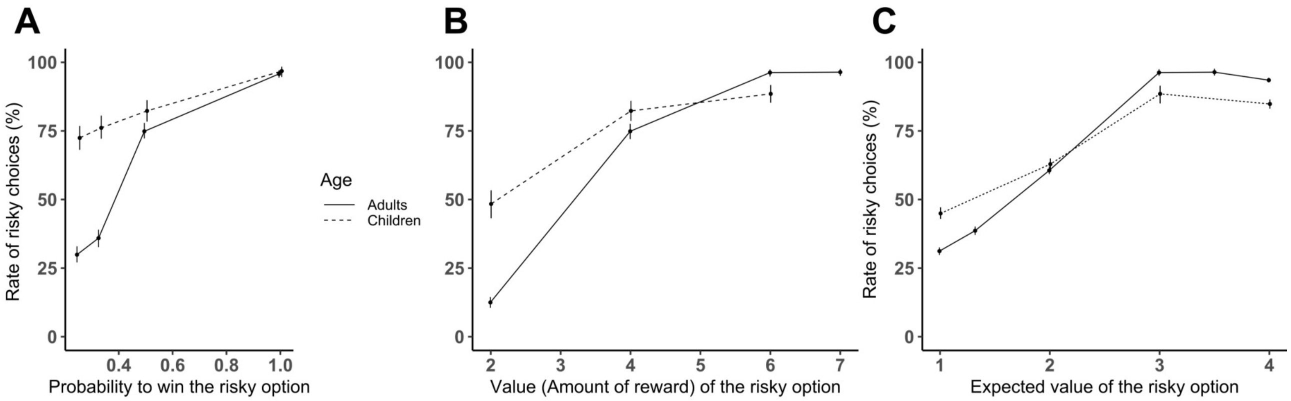

Supplementary Figure 4: Performance comparison between adult and children in Experiment 1.2. Mean percentage of trials where subjects selected the risky option for Experiment 1.1 between adults (solid line) and children (dotted line): (A) according to the value of the risky option (only trials with P=0.5 are considered to allow experiment comparison) and (B) the probability to win (only trials with N=4 are considered) and (C) the expected value (only trials with P=0.5 or N=4 are considered). Error bars indicate 95% confidence intervals.

Supplementary Table 1: Random structure of the full and null models of Study 1. The standard deviations are indicated in the table for the full, the final and null model.

Grouping	Variable	Full model	Null model
Subject	Intercept	0.93	0.65
	Experiment 1.1	0.25	0.45
	Experiment 1.2	1.98	1.31
Block within subject	Experiment 1.1	0.39	0.25
	Experiment 1.2	0.93	0.20

Supplementary Table 2: Estimates the full and null model investigating the impact of experimental and economic, parameters on risky choices in Study 1. The table contains parameter estimates +/- s.e. for the final model. For block, the tested levels are indicated in parentheses.

	Full model	Null model
Intercept	-5.16 +/- 0.16	0.30 +/- 0.08
Sex (male)	- 0.11 +/- 0.17	
Age (child)	2.73 +/- 0.23	
Win probability	4.69 +/- 0.08	
Reward value	0.73 +/- 0.01	
Protocol (1.2)	- 5.29 +/- 0.36	
Win probability : Protocol (1.2)	3.50 +/- 0.30	
Reward value : Protocol (1.2)	1.09 +/- 0.05	
Win probability : Age (child)	- 1.69 +/- 0.14	
Reward value : Age (child)	0.43 +/- 0.02	
Age (child) : Protocol (1.2)	1.93 +/- 0.43	

Supplementary Table 3: Fixed effects of the final model investigating subjects' risky preference in Study 1. The table reports the results of the analysis of deviance (type II Wald chi-square tests).

	Chi-square	Df	p-value
Sex	0.42	1	0.52
Age	3.08	1	0.07
Win probability	3781.02	1	<.001
Reward value	2796.14	1	<.001
Protocol	16.27	1	<.001
Win probability: Protocol	140.15	1	<.001
Reward value: Protocol	447.62	1	<.001
Win probability: Age	136.46	1	<.001
Risky value: Age	316.46	1	<.001
Age: Protocol	19.56	1	<.001

Supplementary Table 4: Power estimates and minimal detectable effect sizes for fixed predictors of the final model of Study 1.

	Size effect (ß)	Power (%)	Minimal detectable size effect (for 80% power)
Sex	- 0.12	15	0.5
Age	2.7	100	0.7
Win probability	4.7	100	0.3
Reward value	0.74	100	0.06
Protocol	- 5.3	100	1

Supplementary Table 5: Random structure of the full and null models of Studies 1 and 2. The standard deviations are indicated in the table for the full, the final and null model.

Grouping	Variable	Full model	Null model
Subject	Intercept	0.58	0.03
	Experiment 1.1	0.14	0.5
	Experiment 1.2	2.05	1.58
	Experiment 2.1	0.72	0.51
	Experiment 2.2	0.96	0.58
Block within subject	Experiment 1.1	0.44	0.27
	Experiment 1.2	0.78	0.05
	Experiment 2.1	0.67	0.28
	Experiment 2.2	0.31	0.09

Supplementary Table 6: Estimates of the full and null model investigating the impact of experimental and economic, parameters on risky choices in Studies 1 and 2. The table contains parameter estimates +/- s.e. for the final model. For block, the tested levels are indicated in parentheses.

	Full model	Null model
Intercept	-5.97 +/- 0.16	-0.008 +/- 0.08
Age (child)	3.91 +/- 0.25	
Win probability	5.55 +/- 0.14	
Reward value	0.76 +/- 0.02	
Protocol (1.2)	-7.8 +/- 0.64	
Protocol (2.1)	-4.06 +/- 0.36	
Protocol (2.2)	-3.8 +/- 0.64	
Win probability : Protocol (1.2)	3.39 +/- 0.5	
Win probability : Protocol (2.1)	2.71 +/- 0.35	
Win probability : Protocol (2.2)	2.95 +/- 0.50	

Reward value : Protocol (1.2)	1.68 +/- 0.10	
Reward value : Protocol (2.1)	0.55 +/- 0.05	
Reward value : Protocol (2.2)	0.61 +/- 0.07	
Win probability : Age (child)	-2.93 +/- 0.21	
Reward value : Age (child)	-0.5 +/- 0.03	
Age (child) : Protocol (1.2)	3.1 +/- 0.61	
Age (child) : Protocol (2.1)	0.15 +/- 0.32	
Age (child) : Protocol (2.2)	0.23 +/- 0.33	

Supplementary Table 7: Fixed effects of the final model investigating subjects' risky preference in Studies 1 and 2. The table reports the results of the analysis of deviance (type II Wald chi-square tests).

	Chi-square	Df	p-value
Age	2.56	1	0.10
Win probability	2890.70	1	<.001
Reward value	1670.35	1	<.001
Protocol	9.30	3	0.025
Win probability : Protocol	121.91	3	<.001
Reward value : Protocol	409.75	3	<.001
Win probability : Age	199.70	3	<.001
Reward value : Age	215.72	1	<.001
Age : Protocol	29.30	1	<.001

Supplementary Table 8: Power estimates and minimal detectable effect sizes for fixed predictors of the final model of Study 2.

	Size effect (ß)	Power (%)	Minimal detectable size effect (for 80% power)
Age	3.91	100	1

Win probability	5.55	100	0.4
Reward value	0.76	100	0.1
Protocol (P2)	-7.8	100	3
Protocol (P3)	-4.06	100	1.5
Protocol (P4)	-3.8	100	1.75

Supplementary Table 9: Estimated slopes of the final model for the economic parameters (probability of the risky option, value of the risky option) in Study 1 and 2.

Trends for the predictor: probability of the risky option

				95% confidence interval		
Age	Experiment	Estimate	Standard error	lower	upper	z-ratio
Adults	1.1	5.55	0.15	5.27	5.84	37.87
	1.2	8.95	0.49	7.99	9.91	18.29
	2.1	8.27	0.35	7.59	8.96	23.72
	2.2	8.51	0.50	7.52	9.49	16.95
Children	1.1	2.63	0.16	2.31	2.95	16.26
	1.2	6.02	0.52	5.01	7.03	11.68
	2.1	5.35	0.36	4.65	6.04	15.02
	2.2	5.58	0.51	4.59	6.57	11.01

Trends for the predictor: value of the risky option

				95% confidence interval		
Age	Experiment	Estimate	Standard error	lower	upper	z-ratio
Adults	1.1	0.77	0.02	0.72	0.81	32.30
	1.2	2.45	0.11	2.24	2.66	23.25
	2.1	1.44	0.06	1.32	1.55	25.04
	2.2	1.38	0.07	1.23	1.52	19.05

Children	1.1	0.27	0.03	0.22	0.32	9.90
	1.2	1.95	0.11	1.74	2.16	18.39
	2.1	0.88	0.07	0.73	1.02	11.83
	2.2	0.94	0.06	0.82	1.05	16.20
